# CD44v6, STn & O-GD2: promising tumor associated antigens paving the way for new targeted cancer therapies

**DOI:** 10.3389/fimmu.2023.1272681

**Published:** 2023-10-03

**Authors:** Iris Lodewijk, Marta Dueñas, Jesus M. Paramio, Carolina Rubio

**Affiliations:** ^1^ Biomedical Research Institute I+12, University Hospital “12 de Octubre”, Madrid, Spain; ^2^ Molecular Oncology Unit, CIEMAT (Centro de Investigaciones Energéticas, Medioambientales y Tecnológicas), Madrid, Spain; ^3^ Biomedical Research Networking Center on Oncology-CIBERONC, ISCIII, Madrid, Spain

**Keywords:** CD44v6, STn, O-GD2, Cancer, Targeted Therapy, O-glycans, Tn, GD2

## Abstract

Targeted therapies are the state of the art in oncology today, and every year new Tumor-associated antigens (TAAs) are developed for preclinical research and clinical trials, but few of them really change the therapeutic scenario. Difficulties, either to find antigens that are solely expressed in tumors or the generation of good binders to these antigens, represent a major bottleneck. Specialized cellular mechanisms, such as differential splicing and glycosylation processes, are a good source of neo-antigen expression. Changes in these processes generate surface proteins that, instead of showing decreased or increased antigen expression driven by enhanced mRNA processing, are aberrant in nature and therefore more specific targets to elicit a precise anti-tumor therapy. Here, we present promising TAAs demonstrated to be potential targets for cancer monitoring, targeted therapy and the generation of new immunotherapy tools, such as recombinant antibodies and chimeric antigen receptor (CAR) T cell (CAR-T) or Chimeric Antigen Receptor-Engineered Natural Killer (CAR-NK) for specific tumor killing, in a wide variety of tumor types. Specifically, this review is a detailed update on TAAs CD44v6, STn and O-GD2, describing their origin as well as their current and potential use as disease biomarker and therapeutic target in a diversity of tumor types.

## 1 Introduction

Immunotherapy, cell therapies, and vaccines are areas of active research and development aimed at harnessing the body**’**s immune system to fight diseases including cancer and infectious diseases. Antigens play a crucial role in these therapies as they elicit an immune response which leads to the activation and targeting of immune cells against specific disease-associated targets.

Tumor-associated antigens (TAAs) are structures expressed by tumor cells that are recognized by the immune system as foreign (1). These antigens can be used as targets for cancer immunotherapies such as adoptive cell transfer (ACT) or cancer vaccines (2, 3). With the growth of technological development and new research techniques every year new TAAs are being developed in order to reach preclinical research and clinical trials, but few of them actually change the therapeutic landscape. Difficulties either to find antigens that are solely expressed in tumors or the generation of good antibodies or specific molecules that recognize these TAAs represent a major bottleneck.

Basic research in specialized cellular machineries such as post-translational modifications (PTMs) and differential splicing is a good source of neo-antigen expression (4–6). Post-translational modifications (PTMs), including phosphorylation, methylation, glycosylation, sialylation and others, introduce structural chemical changes to existing proteins. Even though these proteins are self-proteins, the attachment of an additional glycan or sialyl-group transforms them into foreign antigens. PTMs occurring on tumor cells can therefore give rise to TAAs. Regarding differential splicing, the generation of TAAs can arise from an alternative splicing process during gene expression by which particular exons of a gene are either included or excluded from the final mRNA (7).

Changes in these processes generate surface proteins that, instead of increasing or decreasing protein antigens, as happens with EGFR or other surface proteins driven by increased mRNA processing, produce surface molecules that are aberrant in nature and therefore more specific as targets to elicit a specific anti-tumor therapy. Overall, ongoing research and advancements in understanding the immune system, genomics, and protein engineering are continually expanding the range of potential antigens that can be targeted for immunotherapy, cell therapies, and vaccine development.

It is worth noting that the development and approval of new antigens for immunotherapy, cell therapies, and vaccines involve a complex and rigorous process of preclinical and clinical trials to assess safety and efficacy. Regulatory agencies such as the U.S. Food and Drug Administration (FDA) and the European Medicines Agency (EMA) play a critical role in evaluating and approving these therapies and vaccines (8).

In this review, we introduce a selection of promising TAAs that have shown potential as targets for cancer monitoring, targeted therapy, and the development of new immunotherapy tools. These tools include recombinant antibodies and CAR-T or CAR-NK cells, which can be utilized for precise tumor eradication in various types of tumors. This comprehensive review focuses on the latest information about three specific TAAs: CD44v6, STn, and O-GD2 the first is a variant generated from differential splicing of the well-known CD44 surface marker and the other two are very good examples of cancer associated antigens generated by aberrant glycosylation patterns such as sialylation and O-glycosylation. The review covers their origins, as well as their current and potential applications as disease biomarkers and therapeutic targets across a diverse range of tumor types.

## 2 CD44 alternative splicing as a source of TAAs

**Figure 1:**
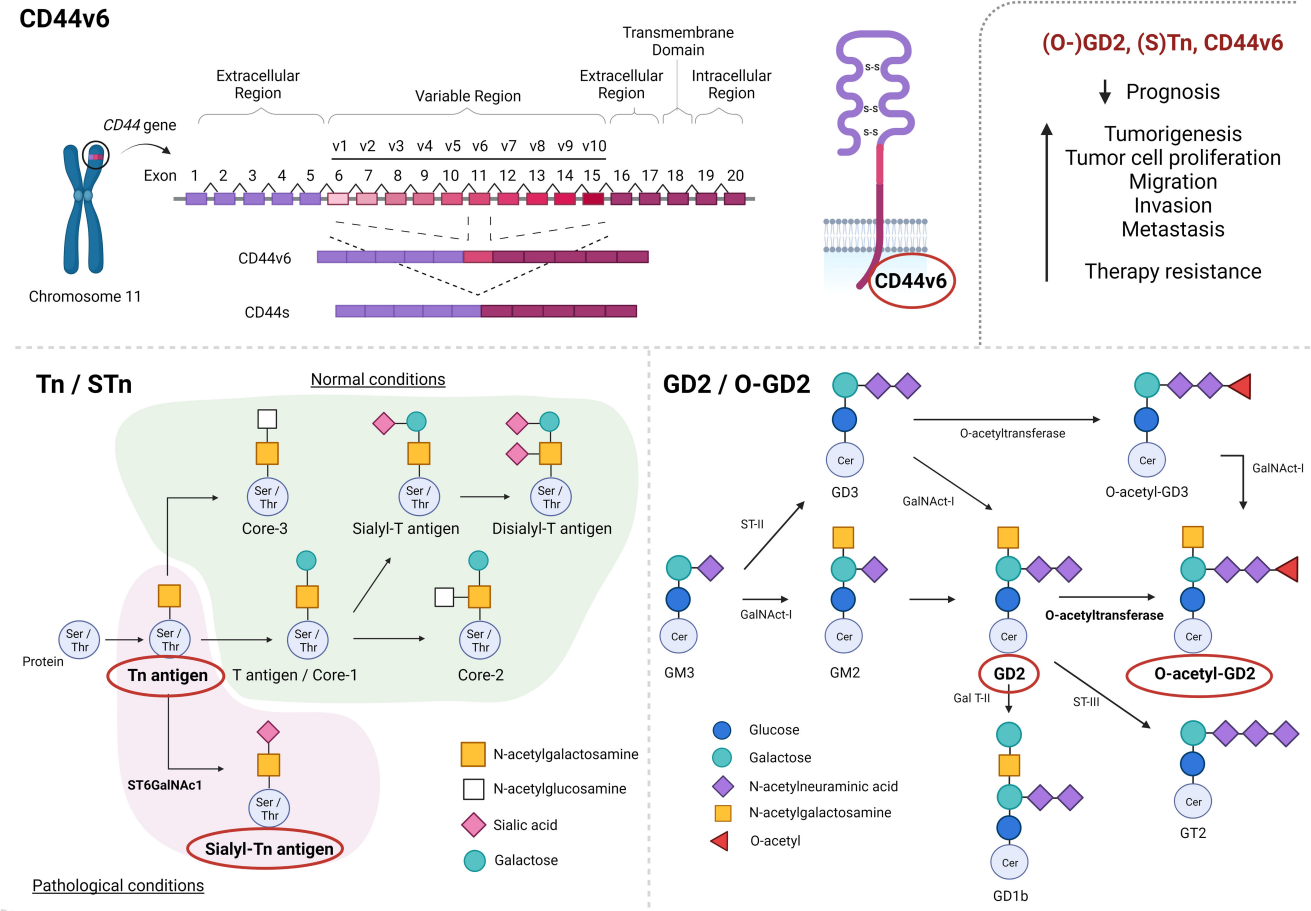
Schematic representation of CD44-alternative splicing, giving rise to CD44 standard form (CD44s) and CD44 isoforms (CD44v1-10), as well as alternative glycosylation processes, generating O-glycans sialyl-Tn (STn) and O-acetyl-GD2 (O-GD2). CD44v6, (S)Tn and (O-)GD2 have been described to negatively impact prognosis as well as to promote tumorigenesis, tumor progression and therapy resistance. Created with BioRender.com.

The role of the CD44 adhesion protein family in neoplastic transformation and invasive potential of carcinomas has been widely considered (9). CD44 represents a complex transmembrane glycoprotein encoded by a single gene on the short arm of chromosome 11 (**Figure 1**, upper). In humans, the CD44 encoding gene consists of 20 exons that give rise to a wide variety of multifunctional glycoproteins due to alternative splicing processes. The standard form, known as CD44s glycopeptide, represents the smallest and most abundant CD44 structure, comprised of exons 1-5 and 16-20. The constant region of the variable isoforms (CD44v) is represented by the same exons, while alternative splicing of the remaining exons (exons 6-15 or CD44v1-10) gives rise to the different CD44v isoforms. It is of note that isoform CD44v1 has not been detected in humans due to the presence of a stop codon in exon 6 (also known as exon v1) (10). The different isoforms of CD44 generated by alternative splicing can act as TAAs and have been implicated in tumor progression and immune evasion. Some CD44 isoforms, such as CD44v6 and CD44v9, have been found to be frequently expressed in many types of cancer, including pancreatic (11, 12), colon (13, 14), and prostate cancer (15, 16). These isoforms are associated with increased invasiveness, metastasis, and resistance to apoptosis.

### 2.1 CD44v6

Alterations of CD44 glycoprotein expression have been shown to play an important role in the progression of various malignancies (17). Specifically, overexpression of the CD44v6 variant has been found in the majority of squamous cell carcinomas and a variety of adenocarcinomas, but has not frequently been observed in non-epithelial tumors (18). In this way, CD44v6 has been described to negatively impact the prognosis of patients with multiple myeloma (19), colorectal cancer (CRC) (20, 21), osteosarcoma (22), esophageal carcinoma (22), gastric cancer (23) as well as head and neck squamous cell carcinoma (HNSCC) (24, 25) patients, among others. Moreover, altered CD44v6 expression has been associated with tumor development, migration, invasion and metastatic potential in a broad variety of tumor types, such as HNSCC (24), oral cancer (26), laryngeal carcinoma (27), esophageal squamous cell carcinoma, gastric cancer (28–30), pancreatic cancer (11, 31), liver cancer (32, 33), CRC (34), lung cancer (35, 36), breast carcinoma (37) and gynecologic malignancies (38) such as ovarian (39, 40) and prostate cancer (41), among others (**Figure 1**).

Circulating tumor cells (CTCs) highly expressing CD44v6 were found in blood samples from metastatic CRC patients (34, 42). Nicolazzo et al. (2020) (43) described treatment failure in those patients with CD44v6-positive CTCs undergoing first-line chemotherapy and proposed that the presence of those cells might be a valuable predictive biomarker of therapy resistance. Indeed, many studies have associated CD44v6 expression with chemoresistance. For example, Gaggianesi et al. (2022) (44) demonstrated that tumor microenvironmental cytokines promote CD44v6 expression in CRC stem cells, conferring resistance to standard anti-tumor therapeutic options, whereas Wang et al. (2019) (45) found that the downregulation of CD44v6 augments chemosensitivity of CRC cells *in vitro*. CD44v6 has been described to enhance CRC therapy resistance by a variety of mechanisms, including activation of the PI3K/Akt/mTOR pathway leading to increased multidrug resistance, up-regulation of the MAPK/ERK pathway stimulating autophagosome formation, and blocking of the Fas-FasL interaction preventing cell apoptosis. Pereira et al. (2020) (46) found similar results, showing that CD44v6+ gastric cancer cells were more resistant to chemotherapy treatment compared to the CD44v6 cells. Even though the exact mechanism in gastric cancer remains unknown, they proposed that therapy resistance might be the result of concomitant CD44v6 expression and either STAT3 or P38 activation, depending on the cellular context. Wang et al. (2021) (45) proposed the involvement of CD44v6 expression in cisplatin resistance in ovarian cancer cells due to the interaction of CD44v6 with P-glycoprotein (P-gp) and, therefore, the acquisition of multidrug resistance. Sagawa et al. (2016) (47) found a correlation between CD44v6 expression and chemoradiotherapy resistance in nasopharyngeal carcinoma, and prostate cancer chemosensitivity was associated with CD44v6 by Ni et al. (2020) (41). Both studies indicated the activation of the PI3K/Akt/mTOR pathway as the major player in the acquisition of therapy resistance. Specifically, in the case of prostate cancer, the interaction between CD44v6 and hyaluronic acid, a component of the extracellular matrix of epithelial and connective tissues, could augment multidrug resistance through up-regulation of the PI3K/Akt/mTOR pathway.

Even though some studies hypothesized the potential role of the majority of CD44 isoforms, such as CD44v3-5 and CD44v8-10, in tumorigenesis and cancer progression, none has been as extensively described as CD44v6 (48). The extensive description of all these different isoforms and their potential implication in tumor development and/or disease progression goes beyond the scope of this review. Beyond alternative splicing process giving rise to different  CD44 isoforms, extensive posttranslational modifications, such as N- and O-glycosylation processes as well as the addition of glycosaminoglycans add another layer of complexity to the CD44 transmembrane glycoprotein structure and function (49).

## 3 STn and O-GD2, the result of alternative glycosylation processes

Altered glycosylation processes are known to be widely involved in tumorigenesis and cancer progression (50). In early cancer stages, the normal synthesis of glycans present in normal epithelial tissues is often impaired due to altered expression of glycosyltransferases, which causes the biosynthesis of truncated carbohydrate structures (such as Tn and STn) which can be observed in a variety of tumor types (49). In the same way, altered ganglioside expression (like GD2 and O-GD2 expression) due to modified glycosyl- and sialyltransferase expression has been found in different cancers such as melanomas and neuroblastomas (51). The role of Tn/STn and GD2/O-GD2 expression in tumorigenesis and cancer progression will be discussed below (**Figure 1**, lower).

### 3.1 Tn & STn

Truncated O-glycan Tn (GalNAcα1-Ser/Thr) and its sialylated variant sialyl-Tn (STn) (Neu5Acα2,6GalNAcα1-Ser/Thr) represent two modifications observed in a variety of proteins which give rise to two tumor-associated antigens that are not expressed in normal cells. Even though O-glycan Tn formation does occur in normal tissue, the Tn glycopeptide represents an immature structure which is normally elongated into other structures by additional glycosyltransferases. Furthermore, altered expression of ST6GalNAc1 glycosyltransferase modifies the Tn glycopeptide by the addition of a sialyl-acid, resulting in STn glycopeptide formation (**Figure 1**). Therefore, the presence of both O-glycan Tn and STn are described to be restricted to pathological conditions, including tumorigenesis (49).

The structural simplicity and biological complexity of the Tn antigen have been extensively reviewed by Ju et al. (2011) (52). Even though the exact role of the Tn antigen in tumor development and progression is only starting to be unraveled, the presence of the Tn antigen on tumor cells was observed as early as 1969 (53). The first correlation between the Tn antigen and cancer was made by Springer et al., who described expression of the Tn antigen in 90% of breast carcinomas. Subsequently, several studies have reported altered levels of the Tn antigen in many tumor types, such as lung, gastric, CRC, bladder, cervical, prostate and ovarian tumors (54–56). Additionally, altered expression of the Tn antigen has been associated with tumor progression, migratory capacity and metastasis as well as poor prognosis in a wide variety of cancer types, including lung cancer (57), pancreatic cancer (58), CRC (59, 60), breast carcinomas and cervical cancer (59, 61).

Similar to the Tn antigen, the presence of the STn antigen has been reported in several tumor types, such as lung cancer, gastric cancer, pancreatic cancer, CRC, breast carcinomas, cervical cancer, prostate and ovarian cancer (62–64). Nevertheless, STn expression in tumors has been described to be rather heterogeneous, with STn-positive cells ranging from 5% to 100% independent of tumor type origin (64). A growing body of evidence also suggests that the role of STn in tumorigenesis might be cancer type-specific and/or organ-specific as STn can be carried by different glycoproteins. Increased expression of STn was associated with cell proliferation and metastasis in gastric, breast and pancreatic cancer (65). In prostate cancer, however, Munkley et al. (2015) (66) described that STn expression is likely to promote cancer cell dissemination and invasion, and this expression is up-regulated in primary prostate carcinoma. Concordantly, Davidson et al. (2000) (67, 68) reported higher levels of STn-positive ovarian cancer cells at the invasive site of primary tumors than in metastatic lesions, and Ferreira et al. (2013) (69) reported increased migratory and invasive capacity of bladder cancer cells upon STn expression. The current hypothesis behind these observations suggests that the STn antigen inhibits tumor formation by reduction of cell-cell and cell-matrix interactions, which facilitates tumor cell spreading. Even though the STn antigen does not seem to provide cell adhesive properties needed for the formation of metastatic lesions, these specific adhesive characteristics are essential for extravasation and invasive capacity. Accordingly, a transient role for STn in cancer progression has been proposed.

Recently, STn expression has also been associated with tumor-microenvironment interactions. Interestingly, a protective role for glycan STn with regard to tumor cell recognition and degradation has been described (70). Either by receptor masking or the inhibition of cytolytic activity, STn has been reported to play an essential role in immunosuppression (71, 72). In bladder cancer, STn expression has been suggested to induce a tolerogenic phenotype in innate and adaptive immune cells. However, extensive studies in a variety of cancer types will be needed to further unravel the role of STn in cancer progression, invasion and metastasis (73).

The STn antigen can be detected in serum when considerable tumor size is reached, either due to O-glycoprotein secretion from tumors or by its expression on CTCs. Therefore, the presence of the STn antigen in serum is thought to be correlated with advanced cancer and, thus, poor prognosis. Indeed, Carvalho et al. (2020) (74) recently reported an association between STn expression and advanced bladder cancer stage and grade. However, the correlation between the STn antigen and prognosis seems to be rather ambiguous and cancer type-specific (64). STn expression was associated with poor prognosis in ovarian cancer (75, 76), but has not been associated with overall survival in either lung (77) or cervical cancer (68). Additionally, contradictory findings have been published for several tumor types, such as breast cancer, esophageal cancer, gastric cancer and CRC, reflecting the potential cancer subtype-specific role of the STn antigen in tumorigenesis and cancer progression (64).

#### 3.1.1 Interaction between CD44v & STn

As mentioned above, STn can be carried by different glycoproteins and glycosylation processes add another layer of complexity to the CD44 glycoprotein. With these observations in mind, CD44 has indeed been reported as carrier protein for STn in gastric and colon cancer. Campos et al. (2015) (78) described the presence of glycan STn on CD44 glycoprotein which leads to altered CD44 molecular features such as molecular weight and antibody recognition in gastric cancer. Mereiter et al. (2019) (79) also reported activation of the receptor tyrosine kinase RON due to increased colocalization of CD44v6 with this receptor in the presence of STn, leading to enhanced hyaluronan binding capacity. Additionally, improved CD44v9 detection by the expression of immature O-glycan structures, such as STn, has recently been proposed by Moreira et al. (2020) (80). Finally, Singh et al. (2001) (81) showed the presence of the STn antigen on CD44 splice variants in CRC, further emphasizing the abovementioned interaction between STn and CD44. Taken together, it might be clear that not only STn or CD44 (v) expression, but also their combination might be very useful with regard to biomarker detection as well as therapeutic targeting.

### 3.2 GD2 & O-GD2

Even though the exact biological mechanisms remain to be elucidated, altered activity of glycosyl- and sialyltransferases seems to be mainly responsible for the modification of ganglioside expression in tumorigenesis (51). For example, increased N-acetylgalactosaminyltransferase I (GM2/GD2 synthase) expression has been found to provoke enhanced GD2 ganglioside levels in melanoma and neuroblastomas (82). Additionally, even though high expression levels of GD2 have been associated with reduced apoptosis as well as enhanced tumor cell proliferation, adhesion, angiogenesis, migration and invasion capacity in a variety of tumor types such as breast cancer (83, 84), bladder cancer (85), lung cancer (86), osteosarcoma (87), Ewing sarcoma (88), retinoblastoma (89) and brain tumors (90), some expression of this ganglioside has been also observed in some normal tissue in healthy adults, like the central nervous system and peripheral nerves (90), Even so, this ganglioside has been used as a biomarker of cancer in serum samples (91) associated with advanced disease and poor prognosis in neuroblastoma by (92), and (85) in high-grade bladder cancer compared to low-grade disease (84). Also, a correlation between GD2 expression and malignant phenotypes of lung cancer has been described by Yoshida et al. (2002) (86) and Esaki et al. (2020) (93).

Even though GD2 expression might be a valuable prognostic marker and biomarker, its expression on healthy cells complicates its potential as therapeutic target (detailed below). Therefore, interest has currently focused on O-acetyl-GD2 (O-GD2), which is formed by the addition of an O-acetyl ester to the GD2 backbone by 9 (7)-O-Acetyl transferase (**Figure 1**, lower right). Fleurence et al. (2017) (51) and Cavdarli et al. (2019) (91) have extensively modeled the complex biosynthetic processes giving rise to the different members of the O-acetylated ganglioside family. Interestingly, O-GD2 has been found to be coexpressed with GD2 on tumor cells. Indeed, the presence of O-GD2 in GD2 positive tumors, such as lung carcinoma, melanoma, osteocarcoma, brain tumors and neuroblastoma, has been confirmed by various studies (94, 95), whereas no expression of O-GD2 was observed in either peripheral nerves or a large variety of other healthy tissues (96).

Even though these findings strongly support O-GD2’s potential as valuable biomarker and therapeutic target, its regulatory mechanisms of expression as well as its role in tumorigenesis remain largely unknown due to the complexity of studying this antigen within the extensive biosynthesis network of the whole ganglioside family. Fleurence et al. (2017) (51) suggested that O-GD2 expression in a cell type depends on the balance and activity of at least 4 different enzymes involved in the biosynthetic model of the ganglioside family.

## 4 CD44v6, STn and O-GD2 as therapeutic targets: past, present and future

### 4.1 CD44v6

**Figure 2:**
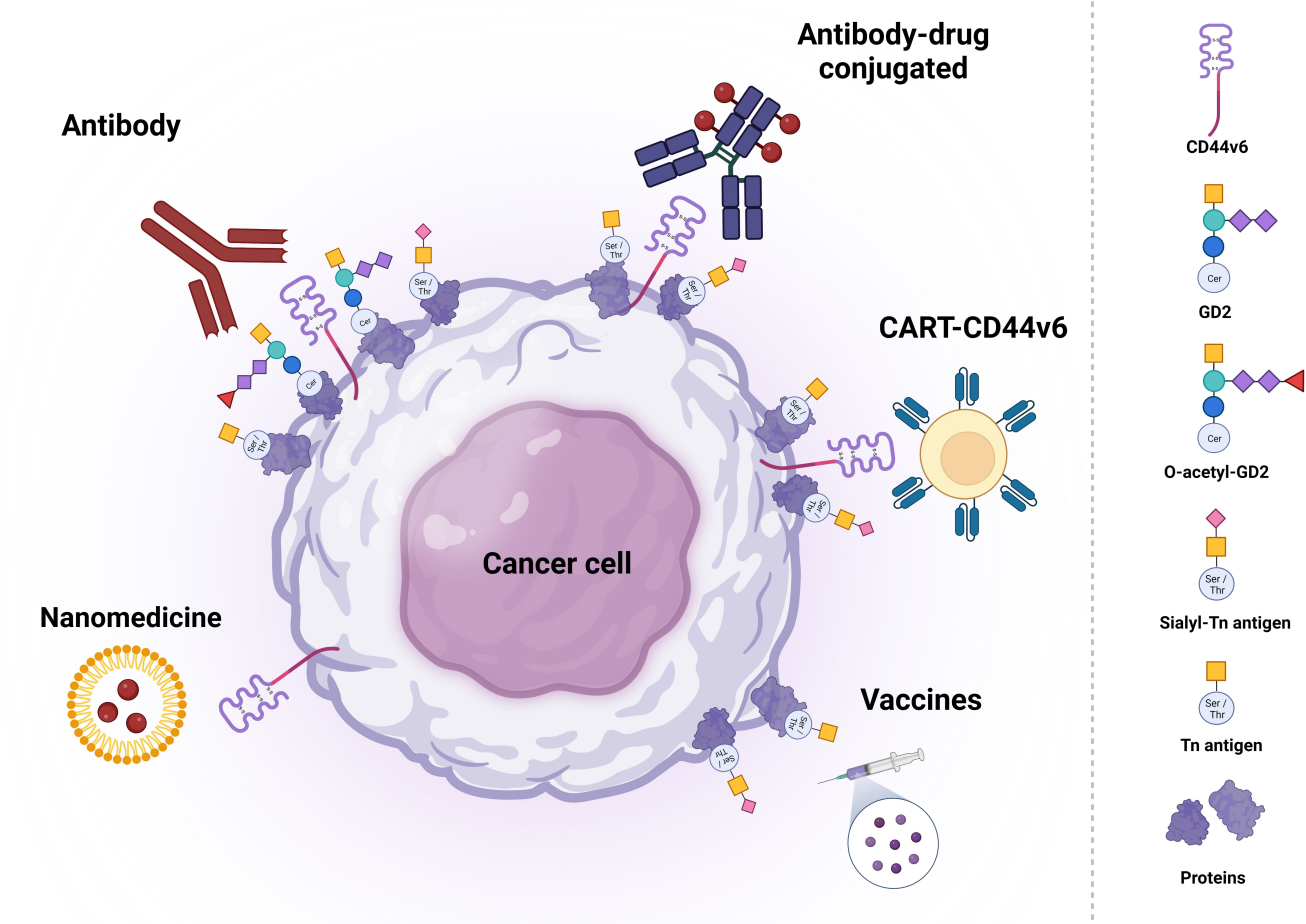
Schematic representation of potential therapeutic strategies based on CD44v6, (S)Tn and (O-)GD2 targeting. Each type of therapy is grouped with different antigens. Created with BioRender.com.

Due to its expression pattern, CD44v6 has been considered an attractive target for antibody-based cancer therapy (17). Thus far, a variety of recombinant antibodies and antibody-drug conjugates have been evaluated in different phase I clinical trials, mainly for their use in HNSCC treatment (96–104). In addition, CD44v6-directed CAR-T cell therapies have shown promising results in preclinical studies, which has led to a phase I/IIa trial in AML and MM patients to study efficacy, safety and feasibility of CD44v6-directed CAR-T cell therapy (105). Even though CAR-T cell therapy for hematological cancers has been very successful, its use for the treatment of solid cancers remains very challenging. Therefore, a variety of studies has evaluated the potential of CD44v6-directed CAR-T cell therapy in solid CD44v6-expressing tumors (105–108). Recently, CD44v6-directed CAR-cytokine induced killer cells (CIK) have demonstrated anti-tumor activity in preclinical studies among different cancer types such as high grade soft tissue sarcomas where the anti-sarcoma activity of CD44v6-CAR-CIK bipotential killers was confirmed in a STS xenograft model in which killing activity was significantly higher compared with unmodified CIK, especially at low effector/target (E/T) ratios: 98% vs 82% (E/T = 10:1) and 68% vs 26% (1:4), (p<0.0001) (109) (**Figure 2**). An extensive overview on the development and potential of the abovementioned immunotherapy strategies can be found below.

#### 4.1.1 Anti-CD44v6 antibody & antibody-drug conjugates

The first studies into the potential of anti-CD44v6 antibody-based immunotherapy were performed almost 30 years ago, when anti-CD44v6 antibodies VFF18 and U36 were characterized as candidates for immunotherapy in squamous cell carcinoma (97–99).

Heider et al. (1996) (97) showed essential therapeutic features for radiolabeled the anti-CD44v6 VFF18 antibody in an *in vivo* model of human epidermoid squamous cell carcinoma. Additionally, a first clinical study was performed by Bree et al. (1995) (98), who demonstrated high and selective tumor uptake for the radiolabeled 99mTc-labeled anti-CD44v6 U36 antibody in HNSCC patients. In agreement with the previous finding, Van Hal et al. (1996) (99) demonstrated that the CD44v6 antigen targeted by anti-U36 is an appropriate target for the treatment of head-and-neck carcinoma by the use of radiolabeled anti-CD44v6 U36 antibody. The biodistribution studies *in vitro*, after which a first phase I clinical trial with 186RE-labeled chimeric antibody U36 among patients with HNSCC showed excellent tumor targeting (100). Even though stable disease as well as reduction in tumor size were observed and the antibody was found to be well-tolerated, myelotoxicity represented a dose-limiting factor. Preclinical characterization of two other radiolabeled anti-CD44v6 antibodies, 111In- and 177Lu-labeled U36 antibodies, using HNSCC *in vitro* and *in vivo* models has also been described as showing a tumor uptake of 72 h.p.i which makes it interesting for therapies (110, 111) cancer detection tool (**Table 1**).

**Table 1:** Overview of the CD44v6 directed therapies described in this study.

Target	Type of therapy	Therapeutic construct	Example	Reference
CD44v6	Anti-CD44v6 antibody & antibody-drug conjugates	Radiolabeled murine/chimeric anti-CD44v6	^99m^Tc-U36	(98)
			^99m^Tc-VFF18 (BIWA1)	(101)
			^186^Re-U36	(100)
			^111^In-U36	(112)
			^177^Lu-U36	(113)
		Radiolabeled humanized anti-CD44v6	^99m^Tc-BIWA4 (bivatuzumab)	(101)
			^186^Re-bivatuzumab	(103, 104)
		Anti-CD44v6 drug conjugate	Bivatuzumab-mertansine	(105, 109, 114, 115)
	CD44v6-CAR-based cell therapy	Anti-CD44v6 CAR-T	scFv 1.1ASML	(19, 107, 116, 117)
		Anti-CD44v6 CAR-CIK		(118, 119)
	CD44v6-based nanomedicine	Anti-CD44v6 conjugated nanoparticle drug delivery	Arsenic trioxide	(120)
			Gemcitabine + siRNA	(121, 122)
			Gemcitabine + miRNA21	(122)
			Bevazucimab	(123)
			MG2477	(124)
		Multi-walled carbon nanotubes (MWNTS)	CXCR4 + gemcitabine	(125)
			CXCR4 + oxaliplatin	(125)
		Polymeric micelles		(126)

This table includes therapeutic target, type of therapy, therapeutic construct and antigenic determinant.

Another phase I clinical study reported high tumor uptake and well-tolerated administration of the murine 99mTc-labeled anti-CD44v6 antibody BIWA1 (initially called VFF18) in patients with HNSCC (101) showing a tumor intake with a mean value of 14.2+/-8.4% of the injected dose/kg tumor tissue and a mean tumor:blood ratio of 2.0+/-1.4 at 40 h after injection with a mean biological half-life in blood (34.5+/-6.1 h). With the aim of clinical application, a humanized 99mTc-labeled anti-CD44v6 antibody BIWA4 antibody, known as bivatuzumab, was then developed, and a phase I clinical trial with HNSCC patients showed selective and high tumor uptake by radioimmunoscintigraphy showing targeting of primary tumors and lymph node metastases in 8 of 10 and 1 of 5 patients, respectively. The highest tumor uptake and tumor to nontumor ratios was observed for the 50-mg dose group with a tumor intake of 26.2 +/- 3.1% of bivatuzumab, without evidence of safety concerns (102). At the same time, phase I clinical studies were performed with HNSCC patients and patients with recurrent or metastatic HNSCC receiving humanized 186Re-labeled bivatuzumab, which showed anti-tumor activity and high tumor to non-tumor targeting ratio (103, 104). Several parallel phase I clinical trials in patients with HNSSC, esophageal carcinoma and metastatic breast cancer were also started with antibody-drug conjugate Bivatuzumab mertansine (105, 109, 114, 115), but the clinical development of this drug was discontinued after a lethal outcome due to toxic epidermal necrolysis in one of the trials. Nevertheless, in 2012, Gurtner et al. (127) reported improved local tumor control at acceptable toxicity levels in an *in vivo* model using lower doses of Bivatuzumab mertansine (the maytansine derivative DM1 as cytotoxic agent) in combination with fractioned irradiation (**Table 1**).

#### 4.1.2 CD44v6-based CARs cell therapy

Other therapeutic strategies based on anti-CD44v6 antibody constructions, which have recently generated interest, include the use of chimeric antigen receptor (CAR) T cell approaches. In 1996, Hekele et al. (110) reported tumor growth suppression in a rat pancreatic adenocarcinoma *in vivo* model when receiving genetically manipulated cytotoxic T lymphocytes (CTLs), expressing the scFv of rat-specific anti-CD44v6 antibody 1.1ASML. The same 1.1ASML antibody was used for the construction of a bispecific F(ab9)2 antibody conjugate (BAC) which recognizes both the CD44v6 tumor cell moiety and the complement receptor CR3 on macrophages in order to redirect and induce efficient macrophage-mediated tumor cytotoxicity. However, *in vitro* and *in vivo* models of the abovementioned pancreatic carcinoma showed lower anti-tumor activity for the BAC compared to the naked anti-CD44v6 1.1ASML antibody (111). So far, promising results regarding CAR-T cell mediated anti-tumor activity have been described for several cancer types such as leukemia (19), multiple myeloma (19, 116), HNSCC (117), lung adenocarcinoma (107) and ovarian cancer (107). In *in vivo* models of acute myeloid leukemia (AML) and multiple myeloma (MM), CD44v6 CAR-T cells were found to provide significant anti-tumor activity without affecting either CD44v6-expressing keratinocytes or hematopoietic stem cells (19). A phase I/IIa clinical trial has subsequently been performed to study safety, efficacy and feasibility of CD44v6 CAR-T cell immunotherapy in AML and MM patients mediating a potent antitumor effects against primary AML and MM while sparing normal hematopoietic stem cells and CD44v6-expressing keratinocytes (105) mediating a potent antitumor effects against primary AML and MM while sparing normal hematopoietic stem cells and CD44v6-expressing keratinocytes (105) mediating a potent antitumor effects against primary AML and MM while sparing normal hematopoietic stem cells and CD44v6-expressing keratinocytes (105) mediating a potent antitumor effects against primary AML and MM while sparing normal hematopoietic stem cells and CD44v6-expressing keratinocytes (105). Additionally, Porcellini et al. (2020) (107) showed CD44v6 CAR-T cell infiltration and proliferation at the tumor site and tumor growth inhibition, leading to enhanced overall survival in xenograft mouse models of ovarian and lung adenocarcinoma with more than 30% of CD44v6-CAR T treated mice still alive after 2 months of observation (106, 117). T cells expressing CD44v6 CAR have been shown to be effective against lung and ovarian adenocarcinomas in mice (107), urothelial carcinoma (106) and pancreatic adenocarcinoma (108). Recently, studies have also focused on optimization of the CAR structure in order to improve CD44v6 CAR-T cell functionality (105) and generating not just CAR-T but CAR-NK cells, which in comparison to CAR-T are proposed to show less cytokine release syndrome associated problems (105) and generating not just CAR-T but CAR-NK cells, which in comparison to CAR-T are proposed to show less cytokine release syndrome associated problems (105) and generating not just CAR-T but CAR-NK cells, which in comparison to CAR-T are proposed to show less cytokine release syndrome associated problemsRaferty et al. (108) demonstrated the efficacy of CD44v6-CAR-NKs in triple negative breast cancer model demonstrating cytotoxic function in both 2D and 3D models of triple-negative breast cancer (108). Nearby to CAR-T and CAR-NK cells, CD44v6-directed CAR-cytokine induced killer cells (CIK) have demonstrated anti-tumor activity in *in vitro* and *in vivo* models of high-grade soft tissue sarcomas such as undifferentiated pleomorphic sarcoma, liposarcoma, fibrosarcoma, leiomyosarcoma and gastrointestinal stromal tumor, among others (118). Moreover, a recent preclinical study proposed the use of CAR-CIK in leukemia patients due to intrinsic anti-tumor activity as well as enhanced safety (119).

#### 4.1.3 CD44v6-based nanomedicine

Finally, CD44v6-targeting nanomedicine has been explored as a promising tool for cancer therapy. Qian et al. (2013) described the use of anti-CD44v6 conjugated nanoparticles carrying the anti-cancer agent Arsenic trioxide in a pancreatic cancer mouse model and its successful accumulation at the tumor site, followed by tumor growth control (120). The same animal model was used for the evaluation of nanoparticle-mediated co-delivery of a chemotherapeutic agent and target genes, either Gemcitabine and small-interfering RNA or Gemcitabine and microRNA-21 (121, 122). Both strategies confirmed CD44v6-directed tumor cell targeting and they were found to provide efficient inhibitory effects on tumor growth and metastasis. Kennedy et al. (2018) (128) reported the specificity and stability of anti-CD44v6 Fab-conjugated poly(lactic-co-glycolic acid (PLGA) nanoparticles *in vivo* in a gastric cancer model, emphasizing its potential as a carrier for cancer therapy in solid tumors, and Baião et al. (2020) (123) described the application of anti-CD44v6 PLGA-PEGylated nanoparticles for bevacizumab delivery in a colorectal cancer model demonstrating intracellular levels of bevacizumab significantly higher in cells incubated with v6 Fab-PLGA-PEG NPs and these nanoparticles resulted in a significant decrease in the intracellular VEGF compared to untargeted nanoparticles and free bevacizumab. In addition, the use of anti-CD44v6 conjugated PEGylated, organic-modified silica (ORMOSIL) nanoparticles carrying the anti-cancer agent 3N-cyclopropylmethyl-7-phenyl-pyrrolo-quinolinone (MG2477) has been shown to provide significantly increased cytotoxic activity towards CD44v6-expressing cells demonstrating that nanoparticles conjugated with a smaller amount of targeting agent being more effective than the ones conjugated with a larger amount of antibody, an effect that will probably be dependent of the affinity of the antigen-antibody interaction (123) demonstrating that nanoparticles conjugated with a smaller amount of targeting agent being more effective than the ones conjugated with a larger amount of antibody, an effect that will probably be dependent of the affinity of the antigen-antibody interaction (123) demonstrating that nanoparticles conjugated with a smaller amount of targeting agent being more effective than the ones conjugated with a larger amount of antibody, an effect that will probably be dependent of the affinity of the antigen-antibody interaction (123) demonstrating that nanoparticles conjugated with a smaller amount of targeting agent being more effective than the ones conjugated with a larger amount of antibody, an effect that will probably be dependent of the affinity of the antigen-antibody interaction (123). Finally, multi-walled carbon nanotubes (MWNTS) and polymeric micelles have been considered as vectors for drug delivery in cancer therapy. Recently, Yin and Qian (2021) (125) showed enhanced anti-tumor activity after treatment with anti-CD44v6 MWNTS, carrying CXCR4 and either gemcitabine or oxaliplatin, in *in vitro* and *in vivo* models of ovarian cancer, whereas Andrade et al. (2021) (126) described the efficacy of anti-CD44v6 polymeric micelles as an anticancer agent carrier (**Table 1**).

Although the above experimental evidences require further proof, the targeted nanoparticle development present clinical potential and provide a launching point for future improvements and therapeutic and/or diagnostic opportunities.

### 4.2 Tn & STn

Based on the restricted expression of O-glycans Tn and STn among human carcinomas, these antigens represent an excellent feature for targeted therapy. As for CD44v6, several recombinant antibodies and antibody-drug conjugates have been evaluated in a variety of phase I/II clinical studies for their anti-tumor efficacy in different tumor types (129–133). Additionally, STn-based CAR-T cell therapy has shown promising results in preclinical studies and, subsequently, reached phase I clinical trials for the treatment of solid tumors (134–136). Nevertheless, the best characterized STn-targeting therapy remains a STn-based vaccine, which has been extensively evaluated in phase III clinical trials (51) (**Figure 2**). An extensive overview on the development and potential of the abovementioned immunotherapy strategies can be found below.

#### 4.2.1 Anti-Tn/STn antibody & antibody-drug conjugates

Many Tn-recognizing antibodies have been generated over the last 35 years. The Tn antigen seems to represent an easy antigenic determinant due to its simple chemical structure (GalNAcα1-Ser/Thr). However, even though Trabbic et al. (2018) (137) published the recognition of a single Tn residue by the anti-Tn Kt-IgM-8 antibody, a single Tn determinant has often been found not be enough for anti-Tn antibody function (138). Accordingly, either the presence of a peptide backbone or multiple consecutive Tn residues has been found to be essential for a wide variety of anti-Tn antibodies. Reis et al. (1998) (89) reported that the PMH1 antibody requires an additional MUC2 apomucin peptide chain, whereas the Tn-MUC1 complex was found to represent the antigenic determinant for several anti-Tn antibodies, including SM3 (139), PankoMab (140) and 5E5 (141). Furthermore, multiple anti-Tn antibodies, such as MLS128 (142), 83D4 (143), KM3413 (144) and Ca3638 (145), have been described to require at least two consecutive Tn residues. Finally, the importance of the Tn backbone composition has been described for some antibodies. For example, Mazal et al. (2013) (146) demonstrated specific recognition of a tri-Serine Tn backbone by the 15G9 antibody (**Table 2**).

**Table 2:** Overview of the (S)Tn directed therapies described in this study.

Target	Type of therapy	Therapeutic construct	Antigenic determinant	Example	Reference
Tn	Anti-Tn antibody & antibody-drug conjugates	Anti-Tn antibody	Tn single residue	Kt-IgM-8	(137)
			Tn-MUC2 complex	PMH1	(89)
			Tn-MUC1 complex	SM3	(139)
				PankoMab	(140)
				5E5	(141)
			Consecutive Tn residues	MLS128	(142)
				83D4	(143)
				KM3413	(144)
				Ca3638	(145)
			Tri-Serine Tn	15G9	(146)
		Humanized anti-Tn antibody	Consecutive Tn residues	cKM3413	(144)
			Tn-MUC1 complex	CIM301-1	(147)
				CIM301-8	(147)
				PankoMab-GEX	(129, 130)
			Consecutive Tn residues	ChiTn	(148)
		Anti-Tn drug conjugate	Consecutive Tn residues	ChiTn - drug conjugate	(148)
		Humanized anti-Tn antibody combination therapy	Tn-MUC1 complex	PankoMab-GEX + anti-EGFR	(131)
	Tn-based vaccines	Anti-Tn vaccine	Tn single residue	MAG-Tn-TT	(149)
	Tn-based CAR-T cell therapy	Anti-Tn CAR-T	Tn-MUC1 complex	Anti-Tn 5E5 CAR-T	(150)
				Anti-Tn SM3 CAR-T	(135, 136)
				Anti-TnMUC1 CAR-T	(151)
STn	Anti-STn antibody & antibody-drug conjugates	Radiolabeled murine anti-STn		^177^Lu-CC49	(152)
				^131^I-CC49	(153)
		Radiolabeled murine anti-STn combination therapy		^131^I-CC49 + IFN	(153)
		Humanized anti-STn antibody		HuCC49ΔCH2	(154)
				^225^Ac-DOTaylated-huCC49	(155)
		Anti-STn drug conjugate		CC49-Br-MMAE	(156)
				SF3-MMAE	(157)
	STn-based vaccins	Anti-STn vaccin		Theratope	(158–162)
	STn-based CAR-T cell therapy	Anti-STn CAR-T		Anti-STn CC49 CAR-T	(134, 163, 164)
		Dual-specific CAR-T		Dual STn- and CD30-specific CAR-T	(165)
				Dual STn- and CD47-specific CAR-T	(166)

This table includes therapeutic target, type of therapy, therapeutic construct and antigenic determinant.

Some of the abovementioned mouse anti-Tn antibodies have been converted into chimeric or humanized antibodies for therapeutic use in the clinic. The mouse-human chimeric antibody, cKM3413, was found to induce ADCC and direct killing activity, which was accompanied by increased survival *in vivo* (144). Additionally, the humanized 5E5 antibodies CIM301-1 and CIM301-8 were reported to enhance NK cell activation and cytotoxicity *in vitro* (147), and humanized PankoMab-GEX was described to be safe, well-tolerated and exhibit promising anti-tumor activity in advanced disease in ovarian cancers, in a phase I clinical trial (129, 130). In 60 evaluable patients with ovarian cancer the clinical benefit included one complete response in a patient treated 483 d and confirmed disease stabilization in 19 patients lasting a median (range) of 23 (10–102) weeks (126). Additionally, the combination of PankoMab-GEX with an anti-EGFR antibody was evaluated in a phase I clinical study and showed anti-tumor activity in lung cancer and CRC patients (131). From this clinical trial were 2 and 4 RECIST partial responses in the first and second part of the study, all in CRC patients. There were 2 responses in each subgroup and the duration of best response was 7.2 months. The PFS for NSCLC was 5.3 months and 2 heavily pretreated patients achieved a prolonged control of disease of 10.6 and 9.4 months. Sedlik et al. (147) has used another mouse-human chimeric antibody, ChiTn, derived from the mouse anti-83D4 antibody, as an ADC for cancer treatment. The ChiTn antibody selectively accumulated in the solid tumor, but not in healthy tissue. When conjugated to saporin (SAP) or to auristatin F, the Chi-Tn ADC exhibited effective cytotoxicity to Tn-positive tumor cells *in vitro* and conjugated to MMAF also induced a delay of tumor growth *in vivo*, validating for the first time the use of an anti-Tn antibody as an effective ADC (148).

The anti-STn antibody CC49 has shown promising. Radiolabeled 177Lu-CC49 was initially demonstrated to provide significant anti-tumor activity *in vivo* (152), which led to its evaluation in phase I and phase I/II radioimmunotherapy clinical trials (132, 133). Here, the 177Lu-CC49 antibody was shown to be well-tolerated and anti-tumor effects were confirmed in patients with chemoresistant ovarian cancer. A differently radiolabeled CC49 antibody, 131I-CC49, was tested in patients with hormone-resistant metastatic prostate cancer. Phase II clinical studies demonstrated enhanced tumor uptake and anti-tumor activity for this antibody in the presence of interferon (IFN) which promoted increased tumor antigen expression and localization compared to the use of the 131I-CC49 antibody alone (153). However, the generation of human anti-murine antibody responses and bone marrow suppression represented two major adverse side-effects, which limited the therapeutic use of these radiolabeled anti-CC49 antibodies. A solution was reported by Rogers et al. (2005) (154) who presented the development of a humanized CC49 antibody, huCC49ΔCH2, and demonstrated less bone marrow toxicity as well as prolonged median survival *in vivo*, probably due to the acceptance of higher radiation doses without limiting side effects and off-side toxicity. In 2021, Minnix et al. (155) described the use of another humanized CC49 antibody construction, 225Ac-labeled DOTaylated-huCC49, in an *in vivo* murine model of ovarian cancer, which showed reduced tumor growth and increased survival without considerable off-side toxicity. Minnix et al. (2020) (156) also proposed an alternative therapeutic strategy using antibody-drug conjugates. The same abovementioned murine model was used to evaluate treatment with the anti-STn antibody-drug conjugate, CC49-Br-monomethyl auristatin E (MMAE, developed from CC49 antibody and monomethyl auristatin E). Reduced tumor growth as well as increased survival were found in mice receiving CC49-Br-MMAE treatment. Finally, another anti-STn antibody-drug conjugate, SF3-MMAE, was reported to be well tolerated and inhibit tumor growth in murine breast and colon cancer models (157) (**Table 2**).

#### 4.2.2 Tn/STn-based vaccines

Another therapeutic strategy is the use of Tn- and STn-based vaccines. In a phase I clinical trial among breast cancer patients with high-risk of relapse, Rosenbaum et al. (2020) (149) evaluated the efficiency of the multiple antigenic glycopeptide-Tn-tetanus toxoid-derived TT830-844 (MAG-Tn-TT) vaccine. This study revealed high levels of Tn-specific antibodies which were found to induce complement dependent cytotoxicity (CDC) and subsequent tumor cell death in all vaccinated patients. It is of note that carbohydrates can induce immune tolerance towards the tumor, which complicates the clinical success of glycan-based cancer vaccines. The development of efficient Tn-based vaccines, such as the MAG-Tn-TT vaccine, might therefore be a complicated process rather than standard therapeutic strategy (167–170).

The same holds for the STn antigen. The generation of an efficient STn-based anti-tumor immunotherapy is challenging due to its poor immunogenicity (71, 171). However, surprisingly, the best characterized STn-targeting therapy is a STn-based vaccine called Theratope. As delayed tumor growth by efficient antibody responses was observed in *in vivo* models receiving Theratope (158, 159), anti-STn immune responses and increased survival were initially also observed in phase I and II clinical studies with Theratope-treated pancreatic, colorectal, breast and ovarian cancer patients (160–162). Nevertheless, phase III clinical trials with metastatic breast cancer patients did not show this overall beneficial anti-tumor activity of Theratope, either alone or in combination treatment with endocrine therapy (172). An extensive review on Theratope´s composition and all clinical studies performed with this STn-based vaccine was published by Julien et al. (2012) (51).

#### 4.2.3 Tn/STn-based CAR-T cell therapy

The construction of anti-STn CAR-T cells and their anti-tumor activity *in vitro* was first described by Hombach et al. (163) in 1997, after which therapeutic benefit of anti-STn CC49-based CAR-T cells was proposed based on *in vivo* models of colon and endometrial carcinoma by McGuinness et al. (164) in 1999. Based on these preliminary findings, several anti-STn CAR-T cell engineering strategies have been evaluated. For example, dual-specific CAR-T cells targeting a second additional antigen, such as CD30 or CD47, have been developed to potentiate the anti-tumor activity of anti-STn CAR-T cells (165, 166). Indeed, Shu et al. (2021) (166) reported delayed tumor growth in low STn expressing tumors when treated with the dual STn- and CD47-specific CAR-T cells, but not after therapy with the single anti-STn CAR-T cells in an *in vivo* ovarian cancer model. It is of note that single anti-STn CAR-T cell therapy was found to be sufficient to obtain the same result in high STn expressing tumors. Finally, the first phase I clinical trials with anti-STn CC49-based CAR-T cells as immunotherapy for solid tumors were published by Hege et al. (134) in 2017. Even though these anti-STn engineered T cells were found to be relatively safe, no considerable clinical efficacy was observed in metastatic CRC patients. Here, rapid clearance of those anti-STn CAR-T cells was proposed as one of the limitations to be overcome for significant therapeutic benefit (**Table 2**).

CAR-T cell strategies targeting Tn or STn have been extensively studied as well. Posey et al. (2016) (150) used the scFv of the abovementioned anti-Tn 5E5 antibody for the development of anti-Tn CAR-T cells, which subsequently showed anti-tumor activity in murine models of pancreatic cancer and leukemia. Anti-Tn CAR-T cells with alternative Tn recognition domains have also been developed and showed therapeutic benefit in *in vivo* models of head and neck cancer (173) and breast and gastric carcinoma (174). In this last case, as an alternative to the use of αβ T cells a subtype of γδ T cells were used, and comparison of both CAR-Ts demonstrated a better cytotoxic effect with the γδ CAR-T cells both *in vitro* and *in vivo*. Additionally, anti-Tn SM3 antibody specificity has been used for the generation of multiple CAR-T constructs, which have been tested in a variety of clinical studies (135, 136). Significant clinical efficacy without evidence of adverse side-effects and safety concerns has been observed in phase I trials including patients with metastatic seminal vesicle cancer (135) and lung cancer (136). Finally, therapeutic efficacy as well as tolerability and safety of anti-TnMUC1 CAR-T cells are being evaluated in a phase I clinical trial among patients with TnMUC1+ multiple myeloma and solid tumors such as lung cancer, breast cancer, pancreatic cancer and ovarian cancer, by Gutierrez et al. (2021) (151). Preliminary efficacy assessed by RECIST v1.1 at Day +28 demonstrate stable disease in all patients that followed lymph depletion chemotherapy.

### 4.3 GD2 & O-GD2

GD2 might be the most relevant glycan in the clinic. Two different antibodies, namely anti-GD2 antibodies 3F8 and 14.18, have been the basis for the generation of clinically successful anti-GD2 therapy (**Figure 2**).

After a variety of phase I clinical studies indicating clinical safety and effectiveness without significant toxicity, either after single or repeated ch14.18 antibody administration (175, 176), a phase II clinical trial showed that the use of ch14.18 combined with granulocyte-macrophage colony-stimulating factor (GM-CSF) and interleukin-2 (IL2) compared to the ch14.18 alone in metastatic neuroblastoma patients was associated with a significantly improved outcome as compared with standard therapy in patients with high-risk neuroblastoma (177). This observation was emphasized by Simon et al. (2004) (178), who demonstrated that the administration of ch14.18 alone did not improve progression-free survival in stage 4 neuroblastoma patients. In 2010, Yu et al. (179) reported a significant increase in event-free survival and overall survival for high-risk neuroblastoma patients treated with a combination of anti-GD2 mAb ch14.18 with IL2 and GM-CSF compared to patients who received standard treatment in a phase III clinical study. Based on these results, Dinutuximab (anti-GD2 ch14.18 antibody) in combination with IL2 and GM-CSF was approved by the United States Food and Drug Administration (FDA) in 2015 for the treatment of high-risk neuroblastoma patients (180). The mechanism of action behind Dinutuximab is based on GD2 binding and subsequent induction of antibody-dependent cell-mediated cytotoxicity (ADCC) as well as CDC, after which recruitment of granulocytes and NK cells finally provoke tumor cell death. In 2017, Dinutuximab β (ch14.18/CHO) was approved by the European Commission for the treatment of high-risk neuroblastoma patients in Europe, whereas Naxitamab (a humanized mAb Hu3F8) in combination with GM-CSF was approved by the FDA in 2020 for the treatment of relapsed high-risk neuroblastoma patients or those who show refractory disease in the bone or bone marrow (181). Currently, several clinical trials are ongoing which will evaluate the potential of Dinutuximab and other hu14.18- and hu3F8-based anti-GD2 antibodies as therapeutic strategies for neuroblastoma and other cancer types including lung cancer, melanoma and osteosarcoma (138, 182). Additionally, alternative therapeutic constructs of anti-GD2 antibodies are being extensively studied, such as radiolabeled antibodies, antibodies modified for drug delivery, GD2-based vaccines, GD2-specific chimeric antigen receptor (CAR) T cells and bispecific antibodies, among others (138, 182). Accordingly, the number of clinical trials evaluating anti-GD2 antibodies for cancer immunotherapy is rapidly growing. An extensive review on approved anti-GD2 antibody-based cancer treatments as well as the many ongoing clinical trials was recently published by Nazha et al. (2020) (182) and Berois et al. (2022) (138).

However, a major bottleneck to the clinical application of anti-GD2-based immunotherapy is the presence of significant adverse side-effects such as neuropathic pain, due to GD2 expression among peripheral nerves (96). In addition to further studies into strategies that circumvent or diminish those significant side effects, alternative approaches for anti-GD2 cancer immunotherapy are being evaluated. Accordingly, the O-GD2 antigen has shown great potential as therapeutic target for cancer immunotherapy due to its absence on healthy tissues, supposing safer therapeutic options and improved treatment tolerance (96). Decades ago Cerato et al. (1997) (183) developed a mouse 8B6-antibody specific for O-GD2 (**Table 3**). Over time, this antibody has been proven to be as efficient as anti-GD2 antibodies regarding the induction of ADCC and CDC (95, 96, 184). Additionally, Cochonneau et al. (2013) (185) proposed a role for the anti-O-GD2 8B6 antibody in tumor cell death by the induction of cell cycle arrest and apoptosis. Even though the exact mechanism behind this observation remains to be elucidated, the apoptosis inducing activity of this O-GD2 antibody might clinically be of major importance, especially in the treatment of tumors that are able to protect themselves from immunological cytotoxicity. For clinical purposes, an alternative mouse/human chimeric version of the 8B6 antibody (c.8B6), maintaining antigen binding affinity and specificity characteristics, was developed by Terme et al. (2014) (184) (**Table 3**). The c.8B6 O-GD2 antibody was found to be as effective as the ch14.18 GD2 antibody, but did not provoke any significant adverse side effects in an *in vivo* model, emphasizing the potentially major clinical benefit of O-GD2-based therapeutic approaches. Nevertheless, whereas many clinical trials with anti-GD2-based antibody therapies are ongoing, clinical trials with an anti-O-GD2 c.8B6 antibody are still awaited.

**Table 3:** Overview of the (O-)GD2-directed therapies described in this study.

Target	Type of therapy	Therapeutic construct	Example	Reference
GD2	Anti-GD2 antibody	Anti-GD2 combination therapy	Dinutuximab (anti-GD2 ch14.18) + IL2 + GM-CSF	(179)
			Naxitamab (anti-GD2 hu3F8) + GM-CSF	(181)
		Anti-GD2 antibody	Dinutuximab β	(180)
		Anti-GD2 radiolabeled antibodies	Dinutuximab and other hu14.18- and hu3F8-based anti-GD2 antibody constructs	(138, 182)
		Anti-GD2 antibodies for drug delivery		
		GD2-based vaccins		
		GD2-specific CAR-T cells		
		Bispecific antibodies		
O-GD2	Anti-O-GD2 antibody	Murine anti-O-GD2 antibody	Anti-O-GD2 8B6 antibody	(183)
		Chimeric anti-O-GD2 antibody	Anti-O-GD2 c.8B6 antibody	(184)

This table includes therapeutic target, type of therapy, therapeutic construct and antigenic determinant.

## 5 Concluding remarks & future perspectives

Taken together, the reviewed antigens represent potential biomarkers and attractive therapeutic targets for personalized medicine in cancer treatment.

The three mentioned TAAs could serve as biomarkers in liquid biopsy and provide valuable information about the presence, progression, and characteristics of tumors. Liquid biopsy involves the analysis of various biomarkers, including circulating tumor cells (CTCs), circulating tumor DNA (ctDNA), and proteins, obtained from a patient’s blood or other bodily fluids (Lodewijk et al., 2018). TAAs can be detected and measured in these samples and offer insights into the underlying cancer biology. Even though some studies have reported the presence of CD44v6, STn and GD2 antigens in serum, no extensive evaluations of their potential as valuable diagnostic, prognostic and/or predictive biomarkers in liquid biopsy have been described. It is of note that the presence of promising biomarker and therapeutic target O-GD2 in serum has not been reported. However, this might be explained by both the recent interest and limited approaches to study the regulatory mechanisms of O-GD2 expression, and further studies into the expression of this antigen are needed to examine the presence of this glycan in serum. Importantly, CD44v6 expression has only been detected on CTCs, whereas STn glycans have been found to be present either on CTCs or as secreted products from tumors. Contrarily, GD2 expression has not been observed on CTCs and is found in the serum as secreted products either from the tumor or exosomes. This observation emphasizes the importance of evaluation of different components of liquid biopsies in order to characterize the presence of newly described antigens in liquid biopsies. Moreover, liquid biopsies not only include serum, but various biological fluids, such as pleural liquid, cerebrospinal fluid, saliva and urine. Therefore, dependent on the cancer type, it might be important to examine CD44v6, STn and O-GD2 expression in other fluids additional to serum. For example, as expression of all those antigens has been observed in lung cancer, the evaluation of pleural liquid might be of great interest. It’s important to note that the choice of TAAs as biomarkers in liquid biopsy depends on the specific type of cancer and the unique genetic alterations associated with it. Different cancers have distinct TAAs, and ongoing research continues to identify and validate novel TAAs for liquid biopsy applications. The utilization of TAAs as biomarkers in liquid biopsy holds promise for non-invasive cancer detection, monitoring, and personalized treatment decisions.

In conclusion, the identification and targeting of tumor-associated antigens have opened up exciting new avenues in cancer treatment. Immunotherapies based on TAAs, such as immune checkpoint inhibitors, cancer vaccines, and CAR-T-cell therapy, have shown great promise in improving patient outcomes. However, it is important to note that the field of TAA-based therapies is still evolving, and further research is needed to optimize treatment strategies, overcome resistance mechanisms, and broaden the application to a wider range of cancer types. Nonetheless, the progress made so far suggests the tremendous potential for the future of cancer treatment.

## Author contributions

CR: Conceptualization, Supervision, Writing – review & editing, Data curation, Validation, Visualization, Funding acquisition, Investigation, Project administration. IL: Conceptualization, Data curation, Investigation, Resources, Writing – original draft. MD: Conceptualization, Data curation, Funding acquisition, Investigation, Supervision, Validation, Visualization, Writing – original draft. JP: Conceptualization, Data curation, Funding acquisition, Investigation, Supervision, Validation, Visualization, Writing – review & editing.
